# The Net Clinical Outcome of Dual-Pathway Inhibition in Clinical Practice: The “Xarelto plus Acetylsalicylic Acid: Treatment Patterns and Outcomes in Patients with Atherosclerosis” Registry

**DOI:** 10.3390/jcm13071956

**Published:** 2024-03-28

**Authors:** Alexander Breitenstein, Alain Gay, Kai Vogtländer, Keith A. A. Fox, Jan Steffel

**Affiliations:** 1 Department of Cardiology, University Hospital Zurich, 8091 Zurich, Switzerland; alexander.breitenstein@usz.ch; 2 University of Zurich, 8006 Zurich, Switzerland; 3 Bayer AG, 13342 Berlin, Germany; 4 Bayer AG, 42096 Wuppertal, Germany; kai.vogtlaender@bayer.com; 5 Centre for Cardiovascular Science, University of Edinburgh, Edinburgh EH16 4TJ, UK; k.a.a.fox@ed.ac.uk

**Keywords:** dual-pathway inhibition, XATOA registry, net clinical benefit

## Abstract

**Background**: In the COMPASS trial, the combination of acetylsalicylic acid (ASA) plus 2.5 mg rivaroxaban twice daily (dual-pathway inhibition, DPI) has been shown to be superior to ASA monotherapy for the reduction in ischemic major adverse cardiovascular events (MACEs, i.e., cardiovascular death, stroke, or myocardial infarction). **Methods**: The international XATOA registry (Xarelto plus Acetylsalicylic acid: Treatment patterns and Outcomes in patients with Atherosclerosis) is a prospective post-approval registry that investigates the cardiovascular outcomes of patients taking ASA plus 2.5 mg rivaroxaban. The aim of this pre-specified analysis was to determine the net clinical outcome (NCO), i.e., a combination of MACEs and bleeding events, of DPI in patients from daily clinical practice. **Results**: Among the 5615 patients, the presence of multiple risk factors resulted in an increase in the total risk of experiencing an NCO event, e.g., from 1.27% (one risk factor) to 2.18% (two risk factors) and 4.07% (three or more risk factors), respectively, with ischemic MACE representing the primary driver of bleeding complications. **Conclusions**: In the real-world XATOA registry, the annual rate of NCO events was low and numerically similar to those seen in the treatment group in the randomized COMPASS trial.

## 1. Introduction

The current pharmaceutical treatment of atherosclerotic vascular disorders focuses on slowing the progression of the disease and preventing cardiovascular events. Nonetheless, patients presenting with chronic coronary syndrome (CCS) or peripheral artery disease (PAD) remain at increased risk of future cardiovascular events even if they adhere to current guideline-recommended treatment. Indeed, even optimized antiplatelet strategies were associated with a relevant residual risk of atherothrombosis in coronary artery disease (CAD) and PAD patients [1,2,3,4], while at the same time substantially increasing the risk of bleeding [5,6,7]. This notwithstanding, antiplatelet therapy has been the standard of care in the setting of CCS and PAD, whereas plasmatic anticoagulation was mainly believed to be important in the prevention of thrombotic events on the low-pressure side of the cardiovascular system, e.g., in venous thromboembolism (VTE) and stroke prevention for atrial fibrillation (AF). However, various lines of evidence have been implying an important role of the plasmatic coagulation system in the high-pressure system of the circulation as well, including in CCS and PAD [8]. While vitamin K antagonists (VKAs) led to a higher risk of bleeding when used for secondary prevention after myocardial infarction, rivaroxaban 2.5 mg twice daily has been shown to reduce the risk of atherothrombotic events in the Anti-Xa Therapy to Lower Cardiovascular Events in Addition to Standard Therapy in Subjects with Acute Coronary Syndrome–Thrombolysis in Myocardial Infarction trial (ATLAS ACS2 TIMI51) and to have an acceptable safety profile when used with single antiplatelet therapy in CAD patients [9,10,11].

Based on this, the double-blind, multicenter COMPASS randomized clinical trial (Cardiovascular Outcomes for People Using Anticoagulation Strategies) was designed [12], enrolling 27,395 high-risk patients with a clinical history of coronary and/or peripheral artery disease (PAD) [13]. The trial showed that the combination of acetylsalicylic acid (ASA) plus 2.5 mg rivaroxaban twice daily (dual-pathway inhibition; DPI) was superior to ASA monotherapy for the reduction in cardiovascular death, stroke, or myocardial infarction [14]. While dual-pathway inhibition led to a higher overall bleeding risk, fatal bleeding and intracranial hemorrhage were not increased. As a result, the net clinical outcome (NCO)—a combination of the most important thrombotic and bleeding events—was strongly in favor of DPI [14]. The NCO is an easy-to-use integrative measure of overall patient benefit as it combines the most important efficacy and safety outcomes (i.e., myocardial infarction, stroke, cardiovascular death, and life-threatening and fatal bleedings). In COMPASS, the predefined NCO was significantly reduced by the combination of rivaroxaban 2.5 mg twice daily plus ASA as compared to ASA monotherapy, primarily by reducing adverse efficacy events (particularly stroke and cardiovascular mortality), whereas severe bleeding events were less frequent and had less clinical impact [13].

One limitation of randomized clinical trials may, however, lie in the potential lack of generalizability of their results to patients encountered in daily clinical practice. The prospective post-approval XATOA registry (Xarelto plus Acetylsalicylic acid: Treatment patterns and Outcomes in patients with Atherosclerosis) was therefore set up to investigate the outcomes of patients on DPI in a non-trial setting [15]. The characteristics of patients in the XATOA registry with CAD and/or PAD are similar to those of the respective patients included in the randomized COMPASS trial [16]. However, in contrast to COMPASS, there was no control arm in the XATOA registry, and as in every prospective, observational registry, a certain selection bias could not be excluded, which is why patients were screened consecutively at the enrolling centers. However, the characteristics of patients included in XATOA are very consistent with the typical CAD population encountered in daily clinical care [17]. The current analysis was performed to assess the NCO of dual-pathway inhibition using rivaroxaban 2.5 mg twice daily (vascular dose) plus acetylsalicylic acid once daily in the XATOA registry in comparison with those observed in the randomized COMPASS trial.

## 2. Methods

The design and methods of the XATOA study have been reported previously [15]. Briefly, XATOA is an international, multicenter, prospective, single-arm registry study in adults ≥ 18 years with either coronary (CAD) or peripheral (PAD) artery disease, or both. Enrolled patients received DPI based on clinical practice decisions. Patients starting DPI within 4 weeks prior to enrollment were included in the XATOA registry. XATOA is a multicenter registry registered at clinicaltrials.gov (NCT03746275). Local ethics committee/institutional board approval was mandatory at all centers and was obtained prior to any patient enrollment. This study complied with the relevant local laws and regulations pertaining to observational studies and data protection and informed consent was obtained from all enrolled patients.

### 2.1. Patient Population and Follow-Up

Patients with either chronic CAD, PAD, or both were enrolled in countries where DPI was approved by local healthcare authorities. The countries included in XATOA were intended to represent a general population of patients where DPI was a possible treatment option at the time of this study. It is important to understand that, although XATOA is related to COMPASS, the eligibility criteria for patients in the XATOA registry were not the same as those of the randomized COMPASS trial. The design of the COMPASS trial as well as the primary outcomes are published elsewhere [12,14]. By virtue of it being a randomized clinical trial, COMPASS had strict inclusion and exclusion criteria for eligibility, as follows: Participants needed to have documented coronary and/or peripheral artery disease, according to the eligibility criteria. In addition, patients with CAD < 65 years of age were eligible only if they had documentation of atherosclerosis involving at least two vascular beds or if they had two additional risk factors (current smoking, diabetes mellitus, an estimated glomerular filtration rate (GFR) < 60 mL per minute, heart failure, or non-lacunar ischemic stroke ≥1 month earlier) [14]. Exclusion criteria included an elevated bleeding risk, recent stroke or previous hemorrhagic or lacunar stroke, severe heart failure, advanced chronic kidney disease (GFR < 15 mL per minute), and the use of dual antiplatelet therapy or full plasmatic anticoagulation [14].

In contrast to the strict inclusion and exclusion criteria of the COMPASS trial, local marketing authorization guided the eligibility of the XATOA registry. The minimum criteria for site selection were the availability of suitable patients and data availability for determining clinical outcomes, as described previously [16]. Patients with contraindications to the locally approved indication for DPI, patients under chronic full-dose oral anticoagulation treatment, and those participating in an interventional trial were excluded. All treatment decisions were at the discretion of the involved clinician. Patients eligible for DPI according to the COMPASS trial were those suffering from PAD or CAD with at least one of the following: (1) age ≥ 65 years, or (2) age < 65 years and atherosclerosis in ≥2 vascular beds or ≥2 additional risk factors (smoking, diabetes, estimated glomerular filtration rate < 60 mL/min/1.73 m^2^, heart failure, or non-lacunar ischemic stroke ≥ 1 month previously).

It was the goal to include patients consecutively at participating sites of XATOA to minimize the risk of selection bias. The follow-up period was at least 12 months after enrollment, and follow-up visits took place according to routine practice. Patients could withdraw from the XATOA study at any time, and substitute patients were not recruited following premature treatment discontinuation. Patients who stopped permanently were followed up regarding their survival status for 30 days after the end-of-study observation by the treating clinician. Data were collected using an electronic data-capturing system. 

### 2.2. Study Outcomes

Clinical outcomes of interest were major adverse cardiovascular events (MACEs), defined as the composite of cardiovascular death, myocardial infarction (MI), or stroke, in line with the COMPASS trial analysis to allow for optimal outcome comparison. The primary safety outcome was ISTH major bleeding, defined as the composite of fatal and/or symptomatic bleeding into a critical organ and/or associated with a ≥2 g/dL reduction in hemoglobin and/or requiring a transfusion of ≥2 units of packed red blood cells or whole blood. The composite endpoint (net clinical outcome; NCO) was defined as a combination of the above-mentioned MACEs and bleeding events (CV deaths, stroke, myocardial infarction, fatal bleedings, or bleedings into a critical organ). Non-major bleeding was defined according to the ISTH criteria (all bleeding events that do not meet the definition of ISTH major bleeding). Adverse events that occurred within 30 days of the last DPI intake were also documented and reported. Subgroup analyses for the net clinical outcome, including specific sub-populations, were pre-specified. Clinical events were adjudicated centrally by an independent external adjudication committee.

### 2.3. Statistical Analysis

The statistical analysis was described earlier [15,16]. All patients who received at least one dose of DPI were added to the final effectiveness analysis, and the safety analysis set was defined as patients who received at least one dose of rivaroxaban. Clinical outcomes were assessed using incidence proportions (with 95% Clopper–Pearson confidence intervals), cumulative incidences, and annualized rates (in patients per 100 patient-years, with 95% Poisson-distributed confidence intervals). Since XATOA was a single-arm prospective study, all statistical analyses were descriptive and exploratory. Clinical outcome measurement was carried out through adverse event reporting (in comparison to COMPASS, where clinical events were captured separately). Comparisons of baseline characteristics were made using Wilcoxon rank-sum test for continuous age and Chi-square test without continuity correction for categorical variables.

## 3. Results

### 3.1. Baseline Characteristics

Patient demographics are summarized in Table 1. A total of 5808 participants were enrolled, of whom 5615 were valid for the analysis (the complete list of exclusion criteria has been previously published) [15]. While participants with an NCO event were significantly older (70.4 ± 9.0 vs. 68.0 ± 9.6 years, *p* = 0.0019), there were no differences in sex (male 79.5% vs. 74.1%, *p* = 0.1138), body mass index (28.2 ± 4.6 vs. 28.2 ± 4.9, *p* = 0.9796), or ethnicity. However, the affected population had a higher prevalence of cardiovascular risk factors, including tobacco use, arterial hypertension (89.8% vs. 80.2%, *p* = 0.0023), or diabetes mellitus (52.4% vs. 38.2%, *p* = 0.0002), as compared to those without an NCO event. In addition, the former suffered more frequently from atherosclerotic vascular manifestations such as coronary (80.1% vs. 72.2%, *p* = 0.0241) and peripheral artery disease (68.1% vs. 58.9%, *p* = 0.0173), previous stroke (17.5% vs. 7.4%, *p* ≤ 0.0001) or myocardial infarction (48.2% vs. 36.1%, *p* = 0.0015), and heart failure (28.9% vs. 16.4%, *p* ≤ 0.0001).

### 3.2. Net Clinical Outcome Events in the Overall XATOA Population

**Figure 1:**
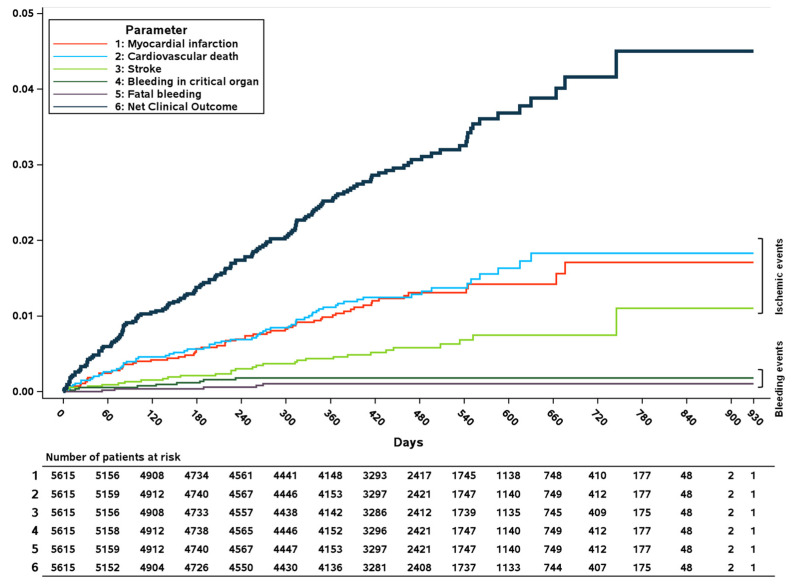
Cumulative incidence of the NCO in the XATOA population.

From the analyzed dataset of 5615 subjects, 158 (2.8% total risk, 2.38%/year, 95% CI 2.02–2.78) experienced at least one treatment-emergent NCO event (Table 2). Figure 1 demonstrates a continuous increase in NCO events over time. The main drivers were MACEs, particularly cardiovascular death and myocardial infarction, while bleeding events only represented a small proportion of NCO events (Figure 1 and Table 2).

### 3.3. Net Clinical Outcome Events in High-Risk Populations

**Figure 2:**
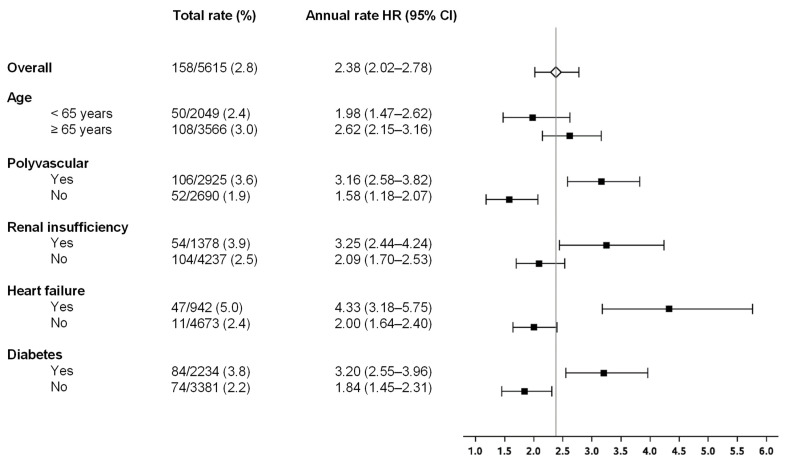
Total and annual rate of net clinical outcome by subgroups in the XATOA population.

**Figure 3:**
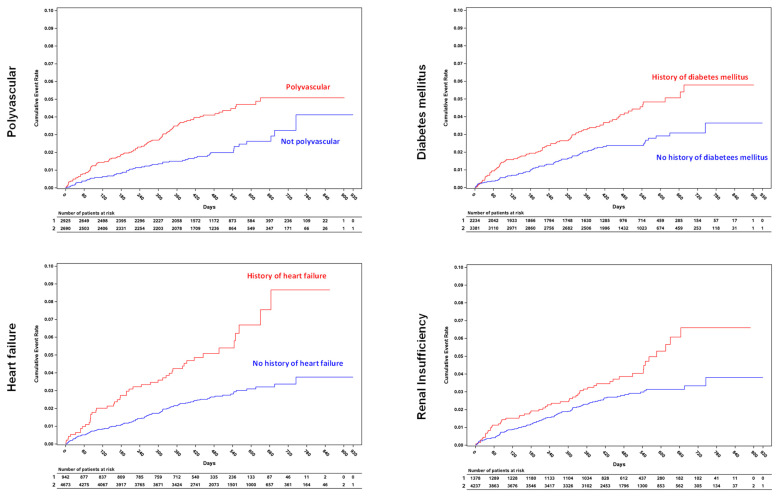
Cumulative incidence of net clinical outcome in patients with either polyvascular disease, heart failure, diabetes mellitus, or renal insufficiency.

The NCO in selected high-risk subjects is shown in Figure 2 and Figure 3. The presence of either polyvascular disease, heart failure, diabetes mellitus, or renal insufficiency was associated with a significantly higher total as well as annual risk of a clinical outcome event (Figure 2 and Figure 3), with heart failure representing the subgroup of patients with the highest risk (total risk of 5%, annual rate 4.33%/yr (95% CI 3.18–5.76), Figure 2 and Figure 3). The Kaplan–Meier curves for NCO events in high-risk subjects separated early in the population suffering from polyvascular disease, heart failure, and diabetes mellitus and continued to separate over time (Figure 3).

**Figure 4:**
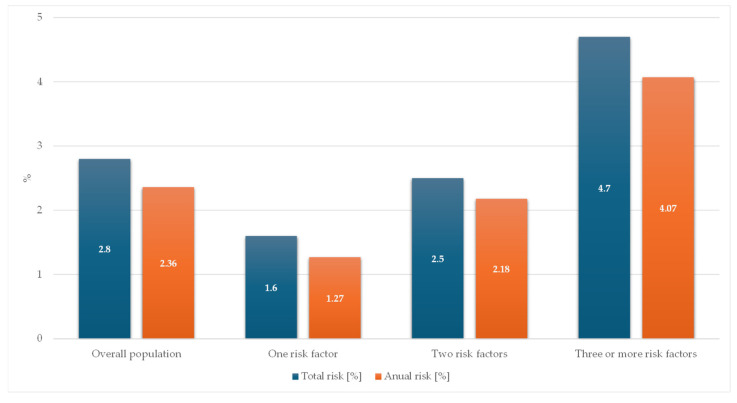
Total and annual risk of net clinical outcome according to the number of risk factors in the XATOA population.

The presence of multiple risk factors had an additive effect on the risk of experiencing a net clinical outcome event. Patients with only one cardiovascular risk factor had a total risk of 1.6% (1.27%/yr), which increased to 2.5% (2.18%/yr) in the population with two risk factors and to 4.7% (4.07%/yr) in those with three or more factors (Figure 4). Further separated by the type of underlying high-risk feature, patients with polyvascular disease, heart failure, or those aged <65 years (who had to have at least two high-risk conditions in order to be eligible for treatment with the DPI regime) had the highest risk of net clinical outcome events.

### 3.4. Comparison of the Results from the XATOA and the COMPASS Populations

**Figure 5:**
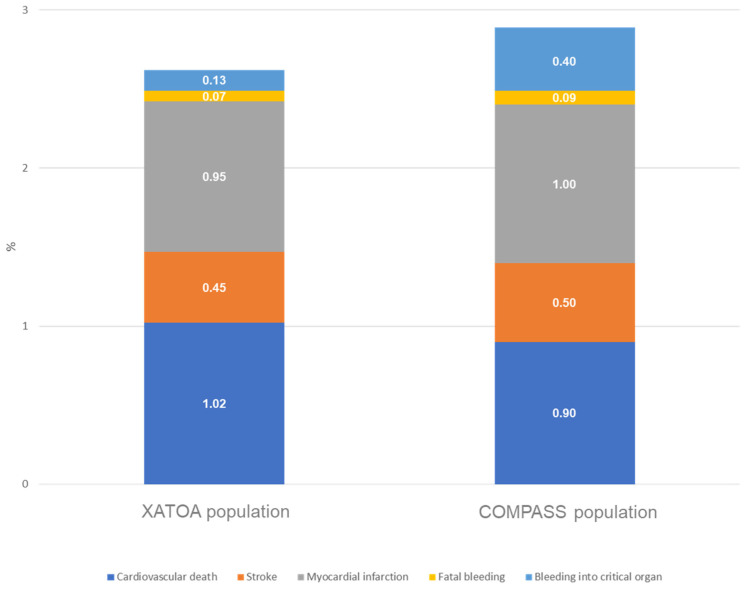
Annual risk of a net clinical outcome event in XATOA and COMPASS.

The proportion of net clinical outcome events was lower in the prospective XATOA registry (2.8%) as compared to the COMPASS trial (4.7%; Figure 5). COMPASS and XATOA had different study durations. However, the annual rate between the XATOA registry and the COMPASS trial population (2.38%/yr vs. 2.5%/yr) as well as the distribution of the components of NCO events were very similar.

## 4. Discussion

XATOA is a prospective international registry conducted in 323 centers from 18 countries to investigate and document the outcome of patients taking DPI in daily clinical practice. The three key findings of the current analysis are the following:

1. Patients treated with DPI in daily clinical practice had annual NCO event rates similar to those of subjects included in the prospective randomized COMPASS trial.
2. As in the COMPASS trial, the NCO event rate was primarily driven by ischemic endpoints and CV death, while the occurrence of severe bleeding events was much less common.
3. The presence of multiple cardiovascular risk factors was associated with an increased risk of an NCO event. Heart failure as a single risk factor had the strongest association with NCO events compared to the other cardiovascular risk factors.

### 4.1. Patient Population in the XATOA Registry

A total of 5808 subjects were enrolled in the XATOA registry, with 5615 having a valid dataset for the current analysis. The previously published prospective, randomized COMPASS trial demonstrated that DPI resulted in a reduction in the primary composite endpoint of cardiovascular death, stroke, and myocardial infarction [14]. Even though the major bleeding rate was 70% higher in the DPI group in COMPASS, the most severe bleeding types were similar, resulting in an NCO in favor of DPI. In comparison to the COMPASS trial, patients enrolled in the XATOA registry had a higher prevalence of hypertension (80.5% vs. 75.5%) and previous stroke (7.7% vs. 3.8%) but had suffered less often from previous myocardial infarctions (36.5% vs. 61.8%). The initiation of DPI in patients included in the XATOA registry was always based on the clinical decision of the treating physician. A previous analysis of the XATOA population confirmed that patients with the highest cardiovascular risk profile as well as those with peripheral arterial disease are being selected for DPI, at least in the context of the registry [16]. This is likely based on the results from the COMPASS trial indicating that patients with diabetes, heart or renal failure, or the presence of polyvascular disease were those with the highest risk of suffering a future cardiovascular event and hence the ones most likely to benefit from DPI [18], which is reflected in the current guidelines on chronic coronary syndromes [19]. Furthermore, not only was the presence of these high-risk cardiovascular conditions similar between XATOA and COMPASS, but the additive value of more risk factors in the same patient (resulting in a higher number of cardiovascular events) was nearly identical in both studies [20].

### 4.2. Contribution of Ischemic and Bleeding Events to the NCO

The predefined NCO endpoints were the same in XATOA and COMPASS, allowing for an indirect comparison of the NCO event rates of the two studies. Moreover, the selection of only the most severe “effectiveness” and “safety” events renders the clinical translation of these results highly meaningful for daily practice as they adequately summarize the most relevant events patients are most afraid of, reducing potential dilution with less severe events. The annual event rate of 2.38%/yr in XATOA was primarily driven by “effectiveness” events, while the rate of severe bleeds was low. The annual event rate was very similar in the XATOA population as compared to COMPASS (2.38%/yr vs. 2.5%/yr, respectively), as was the relative distribution/contribution of individual NCO components. A sub-analysis of the COMPASS data indicated that the NCO event rate steadily increased over time, which was again primarily driven by MACEs rather than bleeding complications [13], and which was nearly identical in XATOA.

Importantly, the observation that the NCO is primarily driven by MACEs in patients treated with DPI cannot be generalized and extended to populations with different anticoagulation regimes. The XANTUS registry, which follows up patients suffering from atrial fibrillation on continuous intake of rivaroxaban 20 mg for stroke and systemic embolization prevention, demonstrated that major bleeding complications are numerically slightly higher than stroke or systemic embolisms (1.9% vs. 0.8%) [21]. The global ETNA-AF registry of atrial fibrillation patients on edoxaban confirms these findings [22]. Even though comparisons between these registries are not directly possible and need to be viewed with care, they underscore that in a patient population suffering from atrial fibrillation under chronic full-dose anticoagulation, bleeding complications may occur to a conceivably higher extent, while in the XATOA population using DPI with a very low dose of rivaroxaban, bleedings are less relevant in comparison to ischemic events.

### 4.3. Effect of Multiple Risk Factors

The presence of multiple risk factors resulted in a steady increase in the risk of experiencing an NCO event, e.g., from 1.27% (one risk factor) to 2.18% (two risk factors) and 4.07% (three or more risk factors), respectively. The presence of polyvascular disease, heart failure, and diabetes was associated with a significant increase in the onset of an NCO event, with heart failure being the strongest associated predictor [14]. In contrast, age (using a cut-off of 65 years) as a single risk factor was not associated with a different risk of an NCO event. Both observations are consistent with the results from the COMPASS trial [13].

The results regarding NCO events were directionally similar in all major subgroups, including chronic kidney disease and heart failure. Also, in these populations, the NCO was primarily driven by ischemic MACEs (including cardiovascular death), whereas severe bleeding events were less common. A detailed analysis of this, however, was beyond the scope of this study and mostly not statistically meaningful due to the relatively low number of outcome events in specific subgroups.

### 4.4. Limitations of the Present Analysis

XATOA is a prospective, observational registry in which, as in other observational studies, a certain selection bias cannot be excluded. To mitigate this risk, patients were screened consecutively at the enrolling centers. This notwithstanding, the characteristics of patients included in XATOA are very consistent with the typical CAD population encountered in daily clinical care [17]. Moreover, reporting of ischemic and bleeding events may be lower than in a controlled phase III trial, and outcomes were not actively evaluated but only captured as adverse events. Another limitation of this single-arm registry is the lack of a control population (including a propensity-matched cohort).

### 4.5. Practical Implications

Our data indicate that the positive data from COMPASS translate into daily clinical practice. As such, patients with chronic coronary syndromes and/or PAD may benefit from the addition of rivaroxaban 2.5 mg twice daily to aspirin in order to prevent future ischemic events [19]. With this additional indication, the adequate selection of the appropriate NOAC for each patient and indication—stroke prevention in atrial fibrillation, treatment/prevention of VTE, etc., and now the prevention of atherothrombotic events in ACS, CAD, and PAD patients—as well as the appropriate dose selection are of paramount importance [23]. The risk of confusion is imminent, particularly since the dose of rivaroxaban studied in COMPASS and XATOA (2.5 mg twice daily) corresponds numerically to the low dose of apixaban approved for stroke prevention in AF. However, in contrast to stroke prevention in AF, where all four NOACs have been tested and approved in most countries, only rivaroxaban 2.5 mg twice daily has been investigated for the prevention of atherothrombotic events in ACS, CAD, and PAD. As such, only this drug and dosing regimen may be used for this indication, and individualization of therapy (i.e., choosing the right NOAC for the right patient) is not a valid possibility. In addition, no dose reduction in the vascular dose of rivaroxaban is applied in the eligible patient population; as the dose of rivaroxaban is already very low, it appears unlikely that inappropriate low-dose use (as it is common in stroke prevention in AF) [23,24] will be similarly prevalent in this indication. Importantly, only patients without an indication for full-dose anticoagulation were included in COMPASS and XATOA; as such, the development of such an indication (e.g., through a new diagnosis of atrial fibrillation) mandates cessation of rivaroxaban 2.5 mg bid—and in most cases of CCS, aspirin—and the initiation of full-dose NOAC therapy.

## 5. Conclusions

In the XATOA registry, patients treated with dual-pathway inhibition using rivaroxaban 2.5 mg twice daily (vascular dose) plus acetylsalicylic acid once daily experienced a numerically low annual rate of NCO events, which was similar to the treatment group of the randomized COMPASS trial. NCO events were primarily driven by MACEs rather than bleeding events.

## Figures and Tables

**Table 1:** Baseline clinical characteristics. Net clinical outcome (NCO) events include CV deaths, stroke, myocardial infarction, fatal bleedings, or bleedings into a critical organ.

	Overall Population (n = 5615)	No Outcome Event (n = 5449)	Outcome Event (n = 166)	*p* Value
**Age, years**	68.1 ± 9.6	68.0 ± 9.6	70.4 ± 9.0	0.0019
**Sex**				
Male	4168 (74.2%)	4036 (74.1%)	132 (79.5%)	0.1138
**Body mass index, kg/m^2^**		28.2 ± 4.9	28.2 ± 4.6	0.9796
**Tobacco use**	n = 5601	n = 5435	n = 166	
Never	1840 (32.9%)	1800 (33.1%)	40 (24.1%)	0.0219
Former	2403 (42.9%)	2329 (42.9%)	74 (44.6%)	
Current	1358 (24.2%)	1306 (24.0%)	52 (31.3%)	
**Hypertension**	4521 (80.5%)	4372 (80.2%)	149 (89.8%)	0.0023
**Diabetes mellitus**	2169 (38.6%)	2082 (38.2%)	87 (52.4%)	0.0002
**Previous stroke**	434 (7.7%)	405 (7.4%)	29 (17.5%)	<0.0001
**Previous myocardial infarction**	2049 (36.5%)	1969 (36.1%)	80 (48.2%)	0.0015
**Heart failure**	942 (16.8%)	894 (16.4%)	48 (28.9%)	<0.0001
**Coronary artery disease**	4066 (72.4%)	3933 (72.2%)	133 (80.1%)	0.0241
**Peripherial artery disease**	3320 (59.1%)	3207 (58.9%)	113 (68.1%)	0.0173
**Estimated GFR**	n = 1556	n = 1508	n = 48	
<30 mL/min	32 (2.1%)	30 (2.0%)	2 (4.2%)	0.0449
30 to 60 mL/min	406 (26.1%)	387 (25.7%)	19 (39.6%)	
>60 mL/min	1118 (71.9%)	1091 (72.3%)	27 (56.3%)	
**Ethnicity**	n = 5313	n = 5151	n = 162	
White	4840 (91.1%)	4687 (91.0%)	153 (94.4%)	
Black	13 (0.2%)	13 (0.3%)	0	0.4711
Asian	339 (6.4%)	332 (6.4%)	7 (4.3%)	
Other	121 (2.3%)	119 (2.3%)	2 (1.2%)	
**Medication at study entry**				
ACE inhibitor or ARB	4087 (72.8%)	3946 (72.4%)	141 (84.9%)	0.0004
Calcium channel blocker	1767 (31.5%)	1701 (31.2%)	66 (39.8%)	0.0196
Diuretic	1707 (30.4%)	1622 (29.8%)	85 (51.2%)	<0.0001
Beta blocker	3469 (61.8%)	3349 (61.5%)	120 (72.3%)	0.0047
Lipid-modifying agent	4835 (86.1%)	4692 (86.1%)	143 (86.1%)	0.9892
Nonsteroidal anti-inflammatory drugs	160 (2.8%)	154 (2.8%)	6 (3.6%)	0.5476
Proton-pump inhibitor	1669 (29.7%)	1608 (29.5%)	61 (36.7%)	0.0445
Treatment for diabetes mellitus, including insulin	1706 (30.4%)	1629 (29.9%)	77 (46.4%)	<0.0001

**Table 2:** Net clinical outcome in the XATOA population and the COMPASS trial. Outcome event rates of the net clinical outcome (CV deaths, stroke, myocardial infarction, fatal bleedings, or bleedings into a critical organ). For comparison, NCOs from the COMPASS trial are added.

	XATOA Population (n = 5615)		COMPASS Population (Rivaroxaban + ASA; n = 9152)	
	Incidence Proportion (n,%)	Annual Event Rate (per 100 p-Years, 95% CI)	Incidence Proportion (n,%)	Annual Event Rate (per 100 p-Years, 95% CI)
Net clinical outcome event (all)	158 (2.8)	2.38 [2.02,2.78]	431 (4.7)	2.5
Cardiovascular death	68 (1.2)	1.02 [0.79,1.29]	160 (1.7)	0.9
Stroke	30 (0.5)	0.45 [0.30,0.64]	83 (0.9)	0.5
Myocardial infarction	63 (1.1)	0.95 [0.73,1.21]	178 (0.9)	1.0
Fatal bleeding	5 (<0.1)	0.07 [0.02,0.17]	15 (0.2)	0.09
Bleeding into critical organ	9 (0.2)	0.13 [0.06,0.26]	78 (0.9)	0.4

## Data Availability

Availability of the data underlying this publication will be determined according to Bayer’s commitment to the EFPIA/PhRMA “Principles for responsible clinical trial data sharing”. This pertains to scope, timepoint and process of data access.
